# A Triple Therapeutic Regiment Consisted of Colchicine, Thalidomide and Total Glucosides of Paeony Is Effective and Well‐Tolerated for Treating Mucocutaneous Involvement in Patients With Behcet's Disease

**DOI:** 10.1002/iid3.70057

**Published:** 2024-12-25

**Authors:** Jian‐Fei Cai, Ya‐Rong Wei, Yong Chen, Jun Zou, Shen Yan, Jian‐Long Guan

**Affiliations:** ^1^ Department of Rheumatology and Immunology Huadong Hospital Affiliated to Fudan University Shanghai China; ^2^ Department of Neurology Xinhua Hospital Affiliated to Shanghai Jiaotong University School of Medicine Shanghai China; ^3^ Shanghai Key Laboratory of Clinical Geriatric Medicine Huadong Hospital Affiliated to Fudan University Shanghai China

**Keywords:** Behcet's disease, colchicine, efficacy, safety, thalidomide, total glucosides of paeony

## Abstract

**Objective:**

This study aimed to investigate the efficacy and safety of a triple therapy consisting of colchicine, thalidomide and total glucosides of paeony (TGP) in Behcet's disease (BD) patients with mucocutaneous involvement.

**Methods:**

Totally 355 newly diagnosed BD patients with mucocutaneous involvement were recruited, who received dexamethasone and colchicine for the first 2 weeks, then they were categorized into “sustained triple‐therapy (ST)” (*n* = 231) and “colchicine to triple‐therapy (CT)” (*n* = 124) groups respectively: for ST group, patients received colchicine, thalidomide plus TGP from Month (M)0.5 to M12; for CT group, patients received colchicine from M0.5 to M2, then switched to colchicine, thalidomide plus TGP from M3 to M12.

**Results:**

The percentages of oral ulceration (at M1, M2) and genital ulceration (at M1) were lower in ST group compared to CT group, whereas there was no difference of other clinical manifestations (including uveitis, erythema nodosum, thrombosis, arterial involvement or nervous system involvement) at each time point between the two groups. For biochemical indexes, ESR was higher at M1 but rapidly reduced at M2 in ST group compared to CT group, while CRP level was similar at all time points between the two groups. For side effects, occurrences of drug‐related cytopenia and diarrhea were increased, in ST group compared to CT group.

**Conclusions:**

A triple therapy consisting of colchicine, thalidomide and TGP is more effective and equally tolerated compared to colchicine alone in treating BD patients with mucocutaneous involvement.

## Graphical abstract

This study aimed to investigate the efficacy and safety of a triple therapy consisting of colchicine, thalidomide and total glucosides of paeony (TGP) in Behcet's disease (BD) patients with mucocutaneous involvement. It showed that A triple therapy consisting of colchicine, thalidomide and TGP is more effective and equally tolerated compared to colchicine alone in treating BD patients with mucocutaneous involvement.

## 1 Introduction

Behcet's disease (BD) is a chronic, relapsing, system‐inflammatory disorder of unknown etiology with a myriad of immunological and pathological consequences [1]. According to global statistics, its highest incidence is found in Turkey (20–420 patients in 100,000 people), also it has a worldwide distribution of 0.12–7.5 per 100,000 in Western countries and 6.3–14 per 100,000 in Eastern countries [2, 3]. Regarding clinical manifestations of BD, it is initially known of the three typical manifestations (including recurrent oral ulcers, genital ulcers and eye disease), while it is currently recognized that BD is a multisystemic disease affecting almost every tissue and organ (such as skin, brain, cardiovascular and central nervous systems), and most of BD patients may suffer an increased risk of recurrence and a high disabling threat, who need a long and sustained treatment process [4, 5]. In view of the multisystemic and recurrent attacks of BD, the persistent efforts to explore effective therapies for BD are of great importance.

For BD management, its goals depend on the site and severity of this disease to alleviate inflammatory attacks as well as relieve BD patients’ symptoms [6, 7]. Colchicine, an anti‐inflammatory plant alkaloid, is known to inhibit neutrophil chemotaxis by inhibiting the microtubule function [8]. And it is the choice for most mild to moderate BD patients with skin and mucosal involvement, which has shown rapid and dramatic efficacy in improving symptoms of erythema nodosum [8]. Currently, one study indicates that colchicine‐corticosteroid treatment might reduce the neutrophil‐to‐lymphocyte ratio in BD patients [9]. Nevertheless, colchicine is unable to effectively control the oral and genital ulcers, thus, applications of more drugs are initiated. As an option for the treatment of patients with resistant severe oral and genital ulcerations, thalidomide has shown its excellent efficacy against oral and genital ulcers, while its clinical application is limited due to its side effects such as sedation, polyneuropathy and teratogenicity [10]. Total glucosides of paeony (TGP), which is a biological compound extracted from *Paeonia lactiflora* pall root, is widely applied in China for multiple diseases, including rheumatoid arthritis (RA), systemic lupus erythematosus (SLE), and liver diseases [11, 12, 13, 14]. Attributing to the anti‐inflammatory, antioxidative and immunoregulatory activities, TGP has achieved remarkable efficacy and good tolerance in these inflammatory diseases, whereas, its application in BD patients has not been reported [11].

Considering no standardization of specific regimen for BD patients has been established due to the high dependence of therapy upon specific manifestations, meanwhile, these above‐mentioned drugs (colchicine, thalidomide and TGP) present with distinct abilities or potentials in relieving inflammatory attacks and symptoms of BD, we hypothesized that a combination regimen consisting of these three drugs might be an effective treatment for BD patients with mucocutaneous involvement. Hence, we proposed a triple therapy consisting of colchicine, thalidomide as well as TGP, and our study aimed to investigate the efficacy and safety of this triple therapy in BD patients with mucocutaneous involvement.

## 2 Materials and Methods

### 2.1 Patients

A total of 355 newly diagnosed BD patients treated in our hospital between January 2017 and December 2017 were recruited in this study. The inclusion criteria were: (i) firstly diagnosed as BD according to the International Criteria for Behcet's Disease (ICBD) [15]; (ii) mainly characterized by mucocutaneous involvement; (iii) without thrombosis, eye involvement, arterial involvement, gastrointestinal involvement, nervous system involvement, or joint involvement at diagnosis; (iv) age more than 18 years old; (v) no contraindications to study drugs; (vi) volunteered to participate in the study and able to be followed up regularly. Patients were excluded if they met any of following criteria: (i) with vital organs involvement that need intensive treatment; (ii) active tuberculosis or complications of a serious infection requiring hospitalization; (iii) history of malignancies; (iv) complicated with other systemic immune system diseases; (v) pregnant or lactating women. The study was approved by the Institutional Review Board of our hospital. All patients provided the written informed consent. The detailed patients' characteristics are presented in Table 1.

**Table 1:** Baseline characteristics of BD patients.

Items	BD patients (*N* = 355)
Age (years), M ± SD	37.5 ± 12.8
Gender, no. (%)	
Female	218 (61.4)
Male	137 (38.6)
Duration of oral ulceration (years), M ± SD	7.1 ± 7.4
History of uveitis, no. (%)	29 (8.2)
Clinical manifestation, no. (%)	
Mucocutaneous involvement	
Oral ulceration	355 (100.0)
Genital ulceration	355 (100.0)
Erythema nodosum	71 (20.0)
Eye involvement	
Uveitis	0 (0.0)
Thrombosis	0 (0.0)
Arterial involvement	0 (0.0)
Nervous system involvement	0 (0.0)
Biochemical indexes, M ± SD	
WBC (×10^9^/L)	5.7 ± 1.5
Neutrophil (×10^9^/L)	56.6 ± 8.0
RBC (×10^12^/L)	3.9 ± 0.5
Hb (g/L)	139.5 ± 13.6
PLT (x10^9^/L)	210.0 ± 58.5
CRP (mg/L)	10.1 ± 9.2
ESR (mm/h)	24.9 ± 18.8
Scr (μmol/L)	61.6 ± 13.2
BUN (mmol/L)	5.2 ± 1.2
ALT (U/L)	21.0 ± 13.2
AST (U/L)	22.9 ± 7.9

Abbreviations: ALT, alanine aminotransferase; AST, aspartate aminotransferase; BD, Behcet's disease; BUN, blood urea nitrogen; CRP, C‐reactive protein; ESR, erythrocyte sedimentation rate; Hb, hemoglobin; M ± SD, mean ± standard deviation; PLT, blood platelet; RBC, red blood cell; Scr, serum creatinine; WBC, white blood cells.

### 2.2 Treatment Process and Regimens

**Figure 1:**
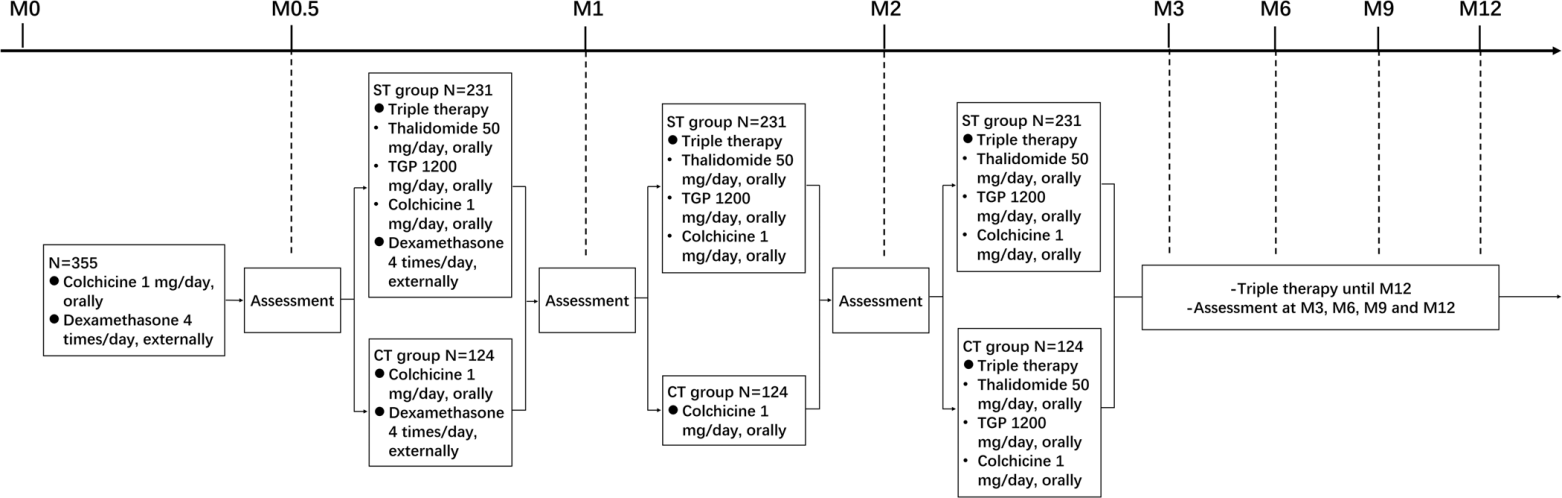
Treatment process.

Detailed treatment process and regimens are shown in Figure 1. After enrollment, all eligible patients received dexamethasone (4 times/day, externally) and colchicine (1 mg/day, orally) for the first 2 weeks. Two weeks later (Month (M) 0.5), according to the disease condition, 231 patients chose to receive the triple therapy with colchicine, thalidomide and total glucosides of Paeonia (TGP), which were divided into “sustained triple‐therapy” group (ST group). While the remaining 124 patients still maintained the initial treatment with dexamethasone for another 2 weeks and colchicine for another 6 weeks, then switched to the triple therapy with colchicine, thalidomide and TGP at M2. These 124 patients were divided into “colchicine to triple‐therapy” group (CT group). For the ST group, treatment process and regimen were as follows: (1) from M0.5 to the end of M1: colchicine 1 mg/day, orally; thalidomide 50 mg/day, orally; TGP 1200 mg/day, orally; dexamethasone 4 times/day, externally; (2) from the end of M1 to the end of M12: colchicine 1 mg/day, orally; thalidomide 50 mg/day, orally; TGP 1200 mg/day, orally. As for the CT group, treatment process and regimen were as follows: (1) from M0.5 to the end of month 1 (M1): colchicine 1 mg/day; dexamethasone 4 times/day, externally; (2) from the end of M1 to the end of M2: colchicine 1 mg/day. (3) from the end of M2 to the end of M12: colchicine 1 mg/day, orally; thalidomide 50 mg/day, orally; TGP 1200 mg/day, orally. During the treatment process, dosage of each drug may fluctuate to some extent, depending on the disease conditions and side effects.

### 2.3 Data Collection and Assessments

The demographic data (age and gender) and the medical history (duration of oral ulceration, history of uveitis) were documented at baseline (M0). The clinical manifestations were assessed at M0, M0.5, M1, M2, M3, M6, M9, and M12. And the biochemical indexes were measured at M0, M1, M2, M3, M6, M9, and M12. The main clinical manifestations were examined as described in the previous study [15], including mucocutaneous involvement (oral ulceration, oral ulceration, and erythema nodosum), eye involvement (uveitis), thrombosis, arterial involvement, and nervous system involvement. The biochemical indexes covered white blood cells (WBC), neutrophil, red blood cell (RBC), hemoglobin (Hb), blood platelet (PLT), C‐reactive protein (CRP), erythrocyte sedimentation rate (ESR), serum creatinine (Scr), blood urea nitrogen (BUN), alanine aminotransferase (ALT), and aspartate aminotransferase (AST). Besides, side effects of study drugs during the study were documented in detail, which included drug‐related cytopenia, drug‐induced diarrhea, drug‐related constipation, drug‐related peripheral nerve injury, drug‐related rash, and drug‐related allergy.

### 2.4 Statistical Analysis

Continuous data were described as mean and standard deviation (SD). Categorical data were described as count and percentage. Student's *t*‐test, Chi‐square test, or Fisher's exact test was used to determine the comparison between two groups. All statistical analyses were performed on the SPSS 22.0 (IBM, USA), and all figures were plotted using the GraphPad Prism 7.01 (GraphPad Prism, USA). *p* value < 0.05 was considered as statistically significant.

## 3 Results

### 3.1 Baseline Characteristics

Totally 355 BD patients (including 218 (61.4%) females and 137 (38.6% males)) were enrolled in this study, with the mean age of 37.5 ± 12.8 years (Table 1). The median duration of oral ulceration was 7.1 ± 7.4 years, and 29 (8.2%) patients had history of uveitis. For the clinical manifestation: 355 (100%), 355 (100%), and 71 (20.0%) patients presented with oral ulceration, genital ulceration and erythema nodosum respectively; while no patients had uveitis, thrombosis or nervous system involvement. As to biochemical indexes, the median values of WBC, neutrophil, RBC, Hb, PLT, CRP, ESR, Scr, BUN, ALT, and AST were 5.7 ± 1.5 (×10^9^/L), 56.6 ± 8.0 (×10^9^/L), 3.9 ± 0.5 (×10^12^/L), 139.5 ± 13.6 (g/L), 210.0 ± 58.5 (×10^9^/L), 10.1 ± 9.2 (mg/L), 24.9 ± 18.8 (mm/h), 61.6 ± 13.2 (μmol/L), 5.2 ± 1.2 (mmol/L), 21.0 ± 13.2 (U/L), and 22.9 ± 7.9 (U/L), respectively.

### 3.2 Clinical Manifestations After 2‐Week Treatment

After 2‐week treatment of colchicine and dexamethasone, 231 (65.1%), 227 (63.9%), 14 (3.9%), 24 (6.8%), 0 (0.0%), 0 (0.0%) and 0 (0.0%) presented with oral ulceration, genital ulceration, erythema nodosum, uveitis, thrombosis, arterial involvement and nervous system involvement, respectively (Table 2).

**Table 2:** Clinical manifestations after two‐week treatment of colchicine and dexamethasone.

Items	Patients (*N* = 355)
Oral ulceration, no. (%)	231 (65.1)
Genital ulceration, no. (%)	227 (63.9)
Erythema nodosum, no. (%)	14 (3.9)
Uveitis, no. (%)	24 (6.8)
Thrombosis, no. (%)	0 (0.0)
Arterial involvement	0 (0.0)
Nervous system involvement, no. (%)	0 (0.0)

### 3.3 Comparison of Clinical Manifestations between ST Group and CT Group

Regarding oral ulceration, its percentage was decreased at M1 (0.0% vs. 21.0%) and M2 (0.0% vs. 100.0%) (both *p* < 0.05), while similar at M3 (0.0% vs. 0.0%), M6 (0.0% vs. 0.0%), M9 (0.0% vs. 0.0%) and M12 (0.0% vs. 0.0%) (all *p* > 0.05) in ST group compared to CT group (Table 3). For genital ulceration, its percentage was reduced at M1 (0.9% vs. 6.5%) (*p* < 0.05), but similar at M2 (0.0% vs. 0.0%), M3 (0.0% vs. 0.0%), M6 (0.0% vs. 0.0%), M9 (0.0% vs. 0.0%) and M12 (0.0% vs. 0.0%) (all *p* > 0.05) in ST group compared to CT group. For erythema nodosum, its percentage was similar at M1 (0.0% vs. 0.0%), M2 (0.0% vs. 0.0%), M3 (0.0% vs. 0.0%), M6 (1.3% vs. 1.6%), M9 (2.6% vs. 4.0%) and M12 (3.5% vs. 2.4%) between ST group and CT group (all *p* > 0.05). Besides, the percentages of other clinical manifestations (including uveitis, thrombosis, arterial involvement and nervous system involvement) were all 0.0% at M1, M2, M3, M6, M9 and M12 in ST group and CT group (all *p* > 0.05). These data indicated that triple therapy was more effective in treating oral ulceration and genital ulceration compared to colchicine alone.

**Table 3:** Comparison of clinical manifestations between CT group and ST group.

Items	CT group (*N* = 124)						ST group (*N* = 231)					
	M1	M2	M3	M6	M9	M12	M1	M2	M3	M6	M9	M12
	No. (%)	No. (%)	No. (%)	No. (%)	No. (%)	No. (%)	No. (%)	No. (%)	No. (%)	No. (%)	No. (%)	No. (%)
Oral ulceration	**26 (21.0)**	**124 (100.0)**	0 (0.0)	0 (0.0)	0 (0.0)	0 (0.0)	0 (0.0)*	0 (0.0)*	0 (0.0)	0 (0.0)	0 (0.0)	0 (0.0)
Genital ulceration	**8 (6.5)**	0 (0.0)	0 (0.0)	0 (0.0)	0 (0.0)	0 (0.0)	2 (0.9)*	0 (0.0)	0 (0.0)	0 (0.0)	0 (0.0)	0 (0.0)
Erythema nodosum	0 (0.0)	0 (0.0)	0 (0.0)	2 (1.6)	5 (4.0)	3 (2.4)	2 (0.9)	0 (0.0)	0 (0.0)	3 (1.3)	6 (2.6)	8 (3.5)
Uveitis	0 (0.0)	0 (0.0)	0 (0.0)	0 (0.0)	0 (0.0)	0 (0.0)	0 (0.0)	0 (0.0)	0 (0.0)	0 (0.0)	0 (0.0)	0 (0.0)
Thrombosis	0 (0.0)	0 (0.0)	0 (0.0)	0 (0.0)	0 (0.0)	0 (0.0)	0 (0.0)	0 (0.0)	0 (0.0)	0 (0.0)	0 (0.0)	0 (0.0)
Arterial involvement	0 (0.0)	0 (0.0)	0 (0.0)	0 (0.0)	0 (0.0)	0 (0.0)	0 (0.0)	0 (0.0)	0 (0.0)	0 (0.0)	0 (0.0)	0 (0.0)
Nervous system involvement	0 (0.0)	0 (0.0)	0 (0.0)	0 (0.0)	0 (0.0)	0 (0.0)	0 (0.0)	0 (0.0)	0 (0.0)	0 (0.0)	0 (0.0)	0 (0.0)

*Note:* Comparison was determined by Chi‐square test or Fisher's exact test. Boldface and *represented *p* value < 0.05.

Abbreviations: CT, colchicine to triple‐therapy group; ST, sustained triple‐therapy group.

### 3.4 Comparison of Biochemical Indexes Between ST Group and CT Group

**Figure 2:**
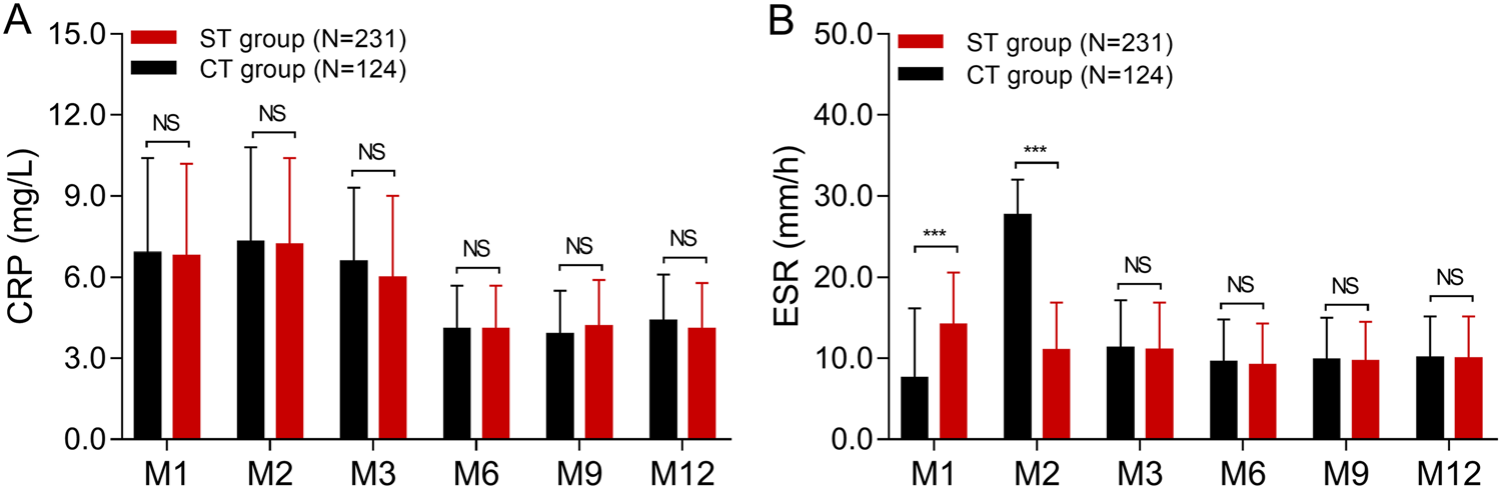
CRP and ESR in ST group and CT group. Comparison of CRP (A) and ESR (B) at different time points between ST group and CT group. CRP, C‐reactive protein; CT group, colchicine to triple‐therapy group; ESR, erythrocyte sedimentation rate; ST group, sustained triple‐therapy group.

For CRP level, it was similar at M1, M2, M3, M6, M9 and M12 between ST group and CT group (all *p* > 0.05) (Figure 2A). For ESR, its value was higher at M1 in ST group compared to CT group (*p* < 0.001), while it rapidly reduced at M2 in ST group compared to CT group (*p* < 0.001), and was similar at M3, M6, M9 and M12 between the two groups (all *p* > 0.05) (Figure 2B), indicating that triple therapy was more effective in decreasing ESR compared to colchicine alone in BD patients.

As to other biochemical indexes at different time points (Table 4): WBC level was increased at M2 (*p* < 0.05), while similar at M1, M3, M6, M9 and M12 (all *p* > 0.05) in ST group compared to CT group; RBC level was decreased at M2 (*p* < 0.05), while similar at M1, M3, M6, M9 and M12 (all *p* > 0.05) in ST group compared to CT group; ALT was elevated at M1 (*p* < 0.05), but similar at M2, M3, M6, M9 and M12 (all *p* > 0.05) in ST group compared to CT group; AST was raised at M1 (*p* < 0.05), but similar at M2, M3, M6, M9 and M12 (all *p* > 0.05) in ST group compared to CT group; no difference of neutrophil, Hb, PLT, Scr or BUN was observed between ST group and CT group at each time point (all *p* > 0.05). Despite some fluctuations of specific biochemical indexes were found between ST group and CT group, the values of all these biochemical indexes remained in the normal ranges.

**Table 4:** Comparison of biochemical indexes between CT group and ST group.

Items	CT group (*N* = 124)						ST group (*N* = 231)					
	M1	M2	M3	M6	M9	M12	M1	M2	M3	M6	M9	M12
	M ± SD	M ± SD	M ± SD	M ± SD	M ± SD	M ± SD	M ± SD	M ± SD	M ± SD	M ± SD	M ± SD	M ± SD
WBC (×10^9^/L)	5.5 ± 1.4	**6.1** ± **1.5**	6.5 ± 1.5	6.6 ± 1.5	6.1 ± 1.5	6.3 ± 1.4	5.6 ± 1.4	**6.5** ± 1.6*	6.3 ± 1.5	6.5 ± 1.7	6.1 ± 1.5	6.0 ± 1.5
Neutrophil (×10^9^/L)	55.0 ± 8.0	61.7 ± 7.5	65.3 ± 5.5	57.8 ± 8.1	63.0 ± 7.6	60.3 ± 8.2	55.3 ± 7.9	62.7 ± 6.9	65.2 ± 5.8	59.2 ± 8.1	61.8 ± 8.1	60.1 ± 8.2
RBC (×10^12^/L)	4.4 ± 0.5	**4.1** ± **0.3**	4.1 ± 0.3	4.0 ± 0.4	4.1 ± 0.4	4.0 ± 0.4	4.4 ± 0.4	**4.0** ± 0.3*	4.1 ± 0.3	4.0 ± 0.4	4.1 ± 0.4	4.0 ± 0.3
Hb (g/L)	138.6 ± 16.6	133.3 ± 9.7	132.5 ± 13.0	134.4 ± 13.2	132.7 ± 11.8	130.7 ± 12.2	139.1 ± 14.0	133.4 ± 10.1	132.6 ± 12.5	133.2 ± 13.9	132.9 ± 12.4	131.3 ± 11.5
PLT (×10^9^/L)	197.3 ± 60.7	218.4 ± 79.4	253.4 ± 89.4	238.6 ± 87.6	260.5 ± 93.4	281.4 ± 96.6	201.5 ± 60.8	210.6 ± 77.7	254.0 ± 82.6	241.7 ± 87.9	275.8 ± 98.7	277.6 ± 93.0
Scr (μmol/L)	66.6 ± 17.9	61.8 ± 18.2	71.8 ± 12.7	61.5 ± 17.5	70.9 ± 13.4	69.0 ± 14.0	65.8 ± 27.1	64.1 ± 18.1	70.4 ± 12.4	62.7 ± 16.3	70.6 ± 14.5	68.1 ± 15.0
BUN (mmol/L)	5.3 ± 1.5	7.0 ± 0.6	6.8 ± 0.6	6.5 ± 0.7	6.6 ± 0.8	6.7 ± 0.6	5.2 ± 1.3	7.0 ± 0.6	6.9 ± 0.6	6.6 ± 0.7	6.5 ± 0.8	6.7 ± 0.6
ALT (U/L)	**18.3** ± **9.6**	28.1 ± 9.4	30.0 ± 10.9	32.3 ± 10.1	29.9 ± 12.1	30.2 ± 10.3	**21.7** ± 13.2*	28.9 ± 10.0	29.5 ± 9.8	32.3 ± 10.6	29.9 ± 11.5	31.6 ± 10.9
AST (U/L)	**20.9** ± **5.5**	27.8 ± 10.1	25.6 ± 9.1	25.6 ± 9.4	25.9 ± 9.6	25.9 ± 8.7	**23.5** ± 9.0*	28.0 ± 10.4	24.6 ± 8.2	26.3 ± 8.7	25.9 ± 9.7	25.5 ± 9.0

*Note:* Comparison was determined by Student's *t*‐test. Boldface and *represented *p* value < 0.05.

Abbreviations: ALT, alanine aminotransferase; AST, aspartate aminotransferase; BUN, blood urea nitrogen; CRP, C‐reactive protein; CT, colchicine to triple‐therapy group; ESR, erythrocyte sedimentation rate; Hb, hemoglobin; M ± SD, mean ± standard deviation; PLT, blood platelet; RBC, red blood cell; Scr, serum creatinine; ST, sustained triple‐therapy group; WBC, white blood cells.

### 3.5 Comparison of Side Effects Between ST Group and CT Group

The occurrence of drug‐related cytopenia was increased at M2 (*p* < 0.05), while was similar at M1, M3, M6, M9 and M12 (all *p* > 0.05) in ST group compared to CT group; the occurrence of drug‐induced diarrhea was elevated at M1, M2 and M3 (all *p* < 0.05), but similar at M6, M9 and M12 (all *p* > 0.05) in ST group compared to CT group; the occurrence of drug‐related constipation was lower at M1, M2, M3 and M6 (all *p* < 0.05), but similar at M9 and M12 (all *p* > 0.05) in ST group compared to CT group; the occurrence of drug‐related peripheral nerve injury was decreased at M6 (*p* < 0.05), but similar at M1, M2, M3, M9 and M12 (all *p* > 0.05) in ST group compared to CT group; the occurrence of drug‐related rash was reduced at M1 (*p* < 0.05), but similar at M2, M3, M6, M9 and M12 (all *p* > 0.05) in ST group compared to CT group; the occurrence of drug‐related allergy was similar at M1, M2, M3, M6, M9 and M12 between ST group and CT group (all *p* > 0.05) (Table 5). These data revealed that triple therapy increased the occurrences of drug‐related cytopenia and drug‐induced diarrhea to some extent, while reduced the occurrences of drug‐related constipation, drug‐related peripheral nerve injury and drug‐related rash compared to colchicine therapy in BD patients.

**Table 5:** Comparison of side effects between CT group and ST group.

Items	CT group (*N* = 124)						ST group (N = 231)					
	M1	M2	M3	M6	M9	M12	M1	M2	M3	M6	M9	M12
	No. (%)	No. (%)	No. (%)	No. (%)	No. (%)	No. (%)	No. (%)	No. (%)	No. (%)	No. (%)	No. (%)	No. (%)
Drug‐related cytopenia	8 (6.5)	**0 (0.0)**	0 (0.0)	0 (0.0)	0 (0.0)	0 (0.0)	14 (6.1)	9 (3.9)*	0 (0.0)	0 (0.0)	0 (0.0)	0 (0.0)
Drug‐induced diarrhea	**0 (0.0)**	**0 (0.0)**	**2 (1.6)**	2 (1.6)	2 (1.6)	0 (0.0)	83 (35.9)*	88 (38.1)*	91 (39.4)*	3 (1.3)	0 (0.0)	1 (0.4)
Drug‐related constipation	**5 (4.0)**	**17 (13.4)**	**20 (16.1)**	**20 (16.1)**	28 (22.6)	23 (18.5)	0 (0.0)*	14 (6.1)*	17 (7.4)*	17 (7.4)*	34 (14.7)	26 (11.3)
Drug‐related peripheral nerve injury	0 (0.0)	0 (0.0)	0 (0.0)	**11 (8.9)**	16 (12.9)	7 (5.6)	0 (0.0)	0 (0.0)	0 (0.0)	6 (2.6)*	17 (7.4)	10 (4.3)
Drug‐related rash	**5 (4.0)**	0 (0.0)	0 (0.0)	4 (3.2)	6 (4.8)	1 (0.8)	0 (0.0)*	0 (0.0)	0 (0.0)	4 (1.7)	5 (2.2)	1 (0.4)
Drug‐related allergy	0 (0.0)	0 (0.0)	0 (0.0)	0 (0.0)	0 (0.0)	0 (0.0)	0 (0.0)	0 (0.0)	0 (0.0)	0 (0.0)	0 (0.0)	0 (0.0)

*Note:* Comparison was determined by Chi‐square test or Fisher's exact test. Boldface and *represented *p* value < 0.05.

Abbreviations: CT, colchicine to triple‐therapy group; ST, sustained triple‐therapy group.

## 4 Discussion

Both colchicine and thalidomide present with outstanding efficacy in achieving adequate control of certain clinical manifestations in BD patients, moreover, TGP has shown great efficacy and well tolerance in various immunological diseases and inflammatory diseases [16, 17, 18, 19, 20, 21, 22, 23, 24]. Thus, we speculated that a triple therapy consisting of these drugs might have good performance in alleviating multiple attacks from BD. Therefore, in our present study, 231 BD patients treated by the sustained triple‐therapy, as well as 124 patients treated by colchicine to triple‐therapy were enrolled, and so we could assess the difference of efficacy between triple therapy and colchicine alone. We observed that triple therapy was more effective in relieving oral ulceration and genital ulceration compared to colchicine alone, and it showed equal ability to improve erythema nodosum as colchicine achieved in BD patients. The following reasons might explain these results: (1) colchicine might interfere with multiple inflammatory processes (such as superoxide production and inflammasome activation) to facilitate attenuating the specific degeneration of the endothelial cells, suppressing neutrophil‐mediated vasculitis, decreasing the vascular stenosis associated with the delayed type hypersensitivity reaction, and reducing lymphocyte‐mediated fat cell lysis and necrosis, which eventually led to alleviated erythema nodosum, thus both the triple therapy and colchicine alone improved the symptom of erythema nodosum in BD patients [8]; (2) thalidomide might ameliorate oral and genital ulcerations through inhibiting neutrophil infiltration and reducing the production of NO and inflammatory cytokines, therefore, the triple therapy involving thalidomide decreased the percentages of oral and genital ulcerations compared to colchicine alone in BD patients [18]; (3) TGP might decrease inflammatory cytokine levels via repressing oxidative stress, which further reduced the inflammatory cell infiltration and edema in mucocutaneous tissues, thus the triple therapy containing TGP had a better performance in attenuating oral and genital ulcerations in BD patients compared to colchicine alone [11, 12]. Besides, triple therapy showed a similar ability in maintaining the normal biochemical indexes as that colchicine alone did (although some fluctuations were observed in triple therapy‐treated BD patients, all the biochemical index levels remained in the normal ranges).

Furthermore, some unavoidable side effects may occur in BD patients treated by common drugs [25, 26]. One study discloses that drug‐related diarrhea happens in 17% BD patients who are treated by colchicine, and another study shows drug‐related nausea and diarrhea happen in 3 out of 8 BD patients who are treated by colchicine [25, 26]. In line with these previous studies, several side effects (including gastrointestinal reactions mentioned in previous studies) were observed in our study. In brief, the triple therapy elevated the occurrences of drug‐related cytopenia and diarrhea to some extent, while considerably decreased the occurrences of drug‐related constipation, peripheral nerve injury and rash compared to thalidomide alone in BD patients. Overall, triple therapy showed equal tolerance with colchicine alone in treating BD patients.

There were some limitations in our study: (1) this was a single‐center study, and some selection biases might exist; (2) patients were not randomized in our study, thus some confounding factors might exist between the two groups and affected the results; (3) patients with thrombosis, eye involvement, arterial involvement, gastrointestinal involvement, nervous system involvement, or joint involvement at diagnosis were excluded to obviate interference, thus our findings might not be applicable for these patients; (4) only newly diagnosed BD patients were enrolled, and our findings in relapsed BD patients required further validations; and (5) this study did not measure the pro‐inflammatory cytokine measurements such as IL‐6 or TNF‐alpha, which should be measured in the further study.

In summary, a triple therapy consisting of colchicine, thalidomide and TGP is more effective and equally tolerated compared to colchicine alone in treating BD patients with mucocutaneous involvement. This study might offer more potential treatment opinions, which would broaden the treatment choice and improve the prognosis of BD patients.

## Author Contributions

**Jian‐Fei Cai:** conceptualization, formal analysis, investigation, methodology, writing–original draft, writing–review and editing. **Ya‐Rong Wei:** conceptualization, formal analysis; methodology, writing–original draft, writing–review and editing. **Yong Chen:** formal analysis, methodology, writing–review and editing. **Jun Zou:** formal analysis, validation, writing–review and editing. **Shen Yan:** investigation, methodology, validation, writing–review and editing. **Jian‐Long Guan:** conceptualization, funding acquisition, methodology, project administration, resources, supervision, writing–review and editing.

## Ethics Statement

The study was approved by the Institutional Review Board of Huadong Hospital Affiliated to Fudan University.

## Consent

All patients provided the written informed consent.

## Conflicts of Interest

The authors declare no conflict of interest.

## Data Availability

The data used or analysed during the current study are available from the corresponding author on reasonable request.
